# Supplementary material for: Splice-Junction-Based Mapping of Alternative Isoforms in the Human Proteome
Source: Cell Rep. Author manuscript; Available in PMC 2020 Jan 15. (PMC6961840; doi:10.1016/j.celrep.2019.11.026)
Supplement: 2 [file NIHMS1546469-supplement-2.zip › DF1/PXD010154/Ovary/Ovary_4_KIAA1217_GPTDEQDACPHECQEQTR.pdf]

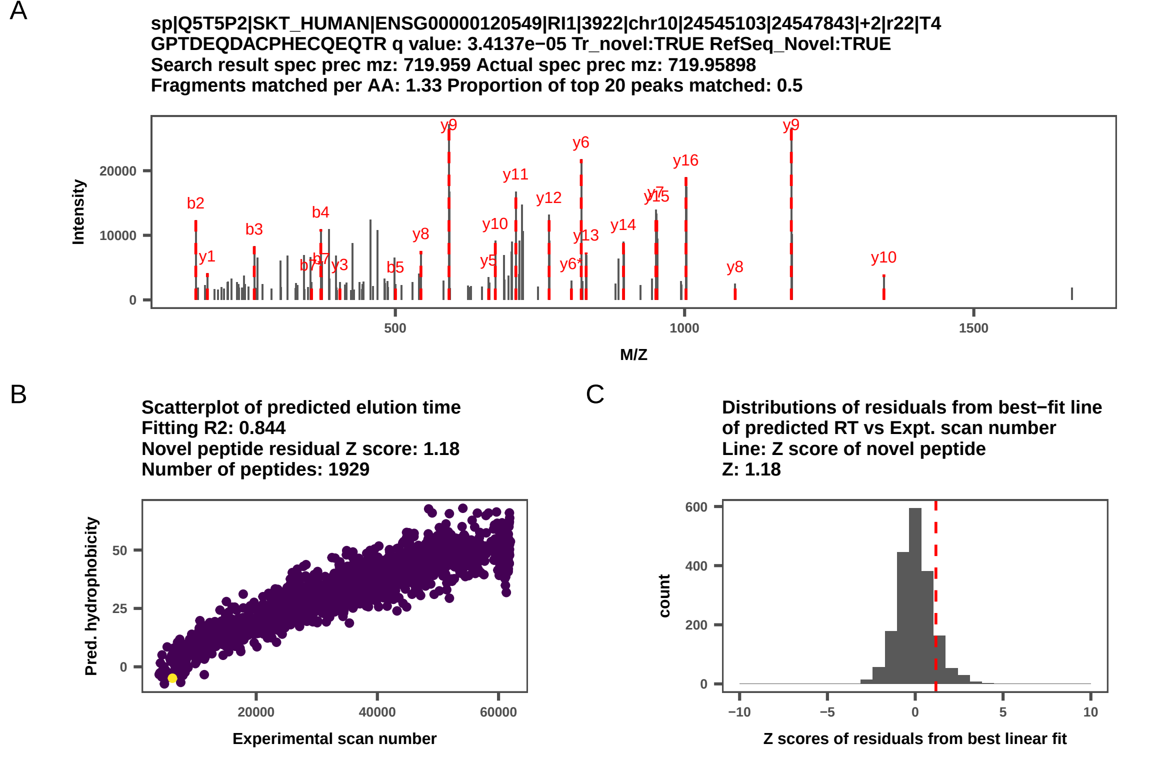
A
sp|Q5T5P2|SKT_HUMAN|ENSG00000120549|RI1|3922|chr10|24545103|24547843|+2|r22|T4
GPTDEQDACPHECQEQTR q value: 3.4137e−05 Tr_novel:TRUE RefSeq_Novel:TRUE
Search result spec prec mz: 719.959 Actual spec prec mz: 719.95898
Fragments matched per AA: 1.33 Proportion of top 20 peaks matched: 0.5
Intensity
0
10000
20000
b2
y1
b3
b7
b7
y3
b4
b5
y8
y9
y5
y10
y11
y12
y6*
y13
y6
y14
y7
y15
y16
y8
y9
y10
500
1000
1500
M/Z
B
Scatterplot of predicted elution time
Fitting R2: 0.844
Novel peptide residual Z score: 1.18
Number of peptides: 1929
Pred. hydrophobicity
0
25
50
20000
40000
60000
Experimental scan number
C
Distributions of residuals from best−fit line
of predicted RT vs Expt. scan number
Line: Z score of novel peptide
Z: 1.18
count
0
200
400
600
−10
−5
0
5
10
Z scores of residuals from best linear fit
